# Odontoid Fractures: A Review of the Current State of the Art

**DOI:** 10.3390/jcm13206270

**Published:** 2024-10-21

**Authors:** Aria Nouri, Michele Da Broi, Adrien May, Insa Janssen, Granit Molliqaj, Benjamin Davies, Naveen Pandita, Karl Schaller, Enrico Tessitore, Mark Kotter

**Affiliations:** 1 Division of Neurosurgery, Geneva University Hospitals, 1205 Geneva, Switzerland; michele.dabroi@hug.ch (M.D.B.); adrien.may@hug.ch (A.M.); insakatrin.janssen@hug.ch (I.J.); granit.molliqaj@hug.ch (G.M.); karl.schaller@hug.ch (K.S.); enrico.tessitore@hug.ch (E.T.); 2 Division of Neurosurgery, University of Cambridge, Cambridge CB2 1TN, UK; bd375@cam.ac.uk (B.D.); naveenpandita1986@gmail.com (N.P.); mrk@cantab.net (M.K.)

**Keywords:** management, surgery, observation, trauma, non-operative treatment, elderly, cervical, peg

## Abstract

Odontoid fractures (OFs) represent up to 15% of all cervical fractures encountered and present most commonly amongst elderly patients, typically in the setting of low energy trauma such as falls. The Anderson and D’Alonzo classification and Roy-Camille subtype description are the most clinically noteworthy descriptions of OFs used. Even though most patients will not present with neurological injury, mechanical instability can occur with type II and type III (Anderson and D’Alonzo) fractures, particularly if the transverse ligament of the atlas is ruptured; however, this is very rare. Conservative treatment is usually employed for type I and type III injuries, and to a varying degree for non-displaced type II injuries. Surgical treatment is typically reserved for type II fractures, patients with neurological injury, and in the setting of other associated fractures or ligamentous injury. Anterior screw fixation is a viable option in the setting of a favorable fracture line orientation in type II fractures, whereas posterior C1–C2 screw fixation is an option for any type II or type III fracture presentation. There is evidence that surgery for type II fractures has higher rates of union and lower mortality than nonoperative treatments. While surgical options have increased over the decades and the management of OF has been optimized by considering fracture subtypes and patient factors, there remains a significant morbidity and mortality associated with OFs. The aging population and changing demographics suggest that there will be an ongoing rise in the incidence of OFs. Therefore, the appropriate management of these cases will be essential for ensuring optimization of health care resources and the quality of life of affected patients

## 1. Introduction

Odontoid Fractures (OF), or fractures of the dens of C2, represent one of the most common types of cervical fractures, and certainly the most common fracture encountered in the upper cervical spine and cranio-cervical junction (CCJ) [1]. These fractures occur frequently amongst the elderly following relatively minor falls [2,3], but can also occur in younger patients, typically from high velocity trauma, such as in motor vehicle accidents [4]. Despite their sensitive anatomical location—adjacent to the upper cervical spinal cord and the lower brainstem—these fractures are not frequently associated with neurological injury [5], unless associated with a significant subluxation.

Given the predominant prevalence of OFs amongst elderly patients, the rising aging population will result in increased encounter rates in clinical practice [3,6]. While this clearly presents a challenge to the health care system, the rise in anticipated cases is further complicated by the controversy surrounding the best strategy for their management [7,8]. Given that OFs vary in type, it is clear that minor fractures (i.e., non-displaced stable fractures, neurologically asymptomatic) are typically treated non-operatively; however, management challenges arise with OFs that are more serious and where clear therapeutic guidance regarding the relative benefits of operative and non-operative management remain unclear.

In an attempt to provide a framework for helping clinicians and surgeons in their decision-making when encountering these fractures, this review seeks to provide the reader with the current state of the art on OFs. For simplicity, fracture types in this article refer to the classification proposed by Anderson and D’Alonzo [9].

## 2. Classification and Imaging

**Figure 1:**
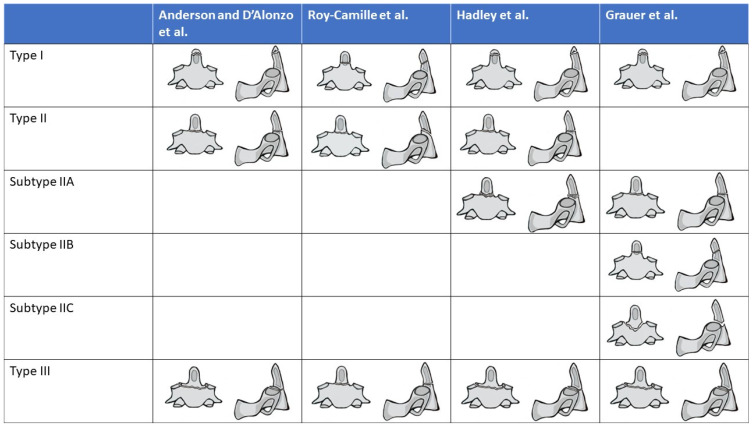
Classification of Odontoid Fractures according to Anderson and D’Alonzo. Sub-classifications of type II fractures according to Roy-Camille, Hadley, and Grauer. Type IV of Roy-Camille classification is not shown in this figure [9,11,12,13].

While an initial classification was proposed by Schatzker et al. in 1970 [10], the most commonly used system for classifying OFs was described by Anderson and D’Alonzo [9] a few years later. Here, the authors separate the fracture into three different types: type I, which are fractures of the tip and above the C1 transverse ligament; type II, which are transverse or oblique fractures through the base of the odontoid (at least one end of the fracture line below the transverse ligament); and type III, which are fractures just below the base of the odontoid which by definition run through the body of the C2 vertebral body (Figure 1). Multiple additional classifications have been proposed in the literature, which focus on modifications of Anderson and D’Alonzo’s description, rather than completely different descriptions, including Hadley [11,12,13], (Figure 1 and Table 1). With regard to the AO Spine classification of upper cervical injuries, all isolated odontoid fractures are considered Type A fractures [14].

## 3. Pathophysiology

Fractures of the odontoid occur almost exclusively due to trauma, though very rarely fractures may occur due to pathologic causes, such as metastatic disease. Typically, low velocity trauma, such as falls, involving the head and neck, which result in sudden hyper-flexion or hyper-extension, are associated with fractures in the elderly with bone fragility. Type II fractures, according to the Anderson D’Alonzo classification, are the most common type given the mechanical vulnerability of the odontoid at its base. This vulnerability of the base is multifactorial: (1) this region is subject to high biomechanical stress, since it is situated between transverse ligaments of C1 above and the C2 vertebral body below; (2) the presence of sub-dental synchondrosis between the dens and C2 body ossification centers, which represents a weak point across the dens [15]; (3) it represents a watershed area, resulting in limited a regenerative capacity and less trabecular bone [16,17], (4) degeneration of the atlanto-odontoid joint and associated local reactive osteosclerosis, increasing C1–C2 rigidity and odontoid bone fragility [2]. While these factors are important, it is also clear that the high level of rotatory mobility of the C1–C2 joint (over 45°) [18] places significant stress on the odontoid, which acts as pivot point. Ultimately, OFs in younger patients are usually the result of high velocity accidents and the abovementioned factors play less of a role. Indeed, younger patients frequently presents with odontoid subluxation and consequently more severe neurological deficit, as well as other cervical spine fractures, particularly C1 fractures.

## 4. Epidemiology

OFs are one of the most common fractures of the upper cervical spine, estimated to represent 9–15% of all cervical fractures [19,20], and occurring more commonly among men in younger cohorts [21,22]. The mean age of injury is not an accurate epidemiological measure, as the occurrence of these fractures is typically bimodal and slanted towards older age, with a smaller peak in young adults (20–30 years of age) typically due to high-speed trauma and a larger peak amongst elderly patients (80 years of age) typically due to falls [3,23]. Due to the nature of their occurrence in the elderly (fragility of bone), it is not surprising that there is substantial increase in the incidence of fractures with increasing age. When looking at C2 fractures in general, an incidence of 0.71/10,000, 2.23/10,000, and 5.67/10,000 person-years has been reported in patients 65–74, 75–84, and over 85 years of age, respectively [3]. OFs are the most common type of fracture occurring at C2 level. OF fractures are also the most common fracture of the atlantoaxial junction, with isolated OFs representing approximately half of the fractures in this region [4]. It has been shown that the rate of incidence has increased over the years and will likely continue to increase with the shifting demographics towards larger cohorts of seniors in many of the developed economies [3,6,23,24].

Large national registry studies have shown that the vast majority of OFs are type II (60–80%) and type III (20–39%), with type I (0–4%) fractures occurring much less frequently [1,23,25,26,27]. As for type II fractures, the most common type of sub-classification (Roy-Camille) appears to be the oblique posterior variant [4] (Figure 1).

## 5. Risk Factors

There are some factors that have been shown to predispose individuals to odontoid fractures. These have been classified into medical and physiological factors, and other factors (such as metastatic disease).

### 5.1. Medical and Physiological

One of the clearest risk factors associated with OFs is age. Multiple studies have shown that the incidence of OFs rises progressively in older adults, with incidence rising with each sequential decade and peaking in the 80s–90s [23,27]. This is likely related to the quality of bone, which becomes more fragile with advancing age. Younger adults who present with odontoid fractures manifest with this fracture due to their propensity for high velocity injuries. The actual factors behind age as a risk factor are likely multiple, including higher risk for low energy falls from other conditions associated with age as well as increased frailty [28]. However, there are also other factors, such as accumulated degenerative changes in the cervical spine that can alter biomechanical forces and flexibility. It has been shown, for example, that significant atlanto-odontoid osteoarthritis in the presence of normal lateral atlantoaxial joints increases the risk of sustaining Type II OFs [2,29]. Others have shown that retro-dens synovitis is more common amongst patients with OF [30].

The fragility of bone, due to osteoporosis, is another important contributor predisposing to OFs [29,31], and it has been shown that the cervical bone mineral density is an independent significant risk factor for odontoid fractures but not for other fractures of the cervical spine [32]. It has also been shown that odontoid cysts predispose one to OF [30], and specifically Type II OF factures [32]. This fragility can also predispose an individual to pseudoarthrosis with both conservative management or surgical fixation [33].

Bone fragility may also arise from other conditions such as rheumatoid arthritis and ankylosing spondylitis [34,35,36]. While patients with ankylosing spondylitis present with OFs due to low energy trauma, patients with rheumatoid arthritis typically present with atraumatic fractures. The risk associated with ankylosing spondylitis is related to the rigidity and brittleness of the fused spine, which leads to the spine behaving much like an osteoporotic long bone rather than an elastic spine [34]. Moreover, given that ankylosing spondylitis typically spares the cranio-cervical junction and rigidifies the rest of the spine, the C1–C2 region becomes a transitional area which undergoes an increased biomechanical stress. The reason behind atraumatic fractures amongst patients with rheumatoid arthritis is less well known.

### 5.2. Other Rare Risk Factors

A rare but important risk factor for OF is metastatic disease of the C2 vertebra or an odontoid peg leading to a pathologic fracture. Such cases may not be associated with trauma, but due to pain are often treated with a combination of vertebroplasty and stabilization [37,38].

Rarer risk factors for OF include congenital malformations of the cervical region, particularly in the C1–C2 region, which can alter the biomechanics and stress forces related to the odontoid [39]. While many such congenital conditions (Klippel–Feil Syndrome, Down’s Syndrome) exist, literature more substantial than case reports that discuss this topic is practically non-existent. One exception is a small report suggesting that *ponticulus posticus* anomaly may increase the risk for Type II OFs [40].

It is important to note that while other notable developmental anomalies of the odontoid exist, such as *os odontoideum*, persistent ossiculum terminale, and odontoid aplasia, these have not been shown to predispose individuals to OF, but may, however, increase atlantoaxial instability [41,42].

## 6. Clinical Presentation

The typical presentation of OF is an elderly patient who arrives at the emergency department after an accidental fall from standingand who presents with neck pain, particularly with movement. The other less common group of individuals who present are patients having sustained high energy trauma such as from a motor vehicle accident; these OFs are, however, typically associated with multiple other injuries. Despite suffering from mechanical instability, neurological injury is present in only a minority of patients, with most studies reporting an incidence of ≤13% [5,43,44], and a single smaller and older study reporting a rate of 29% [45]. SCI in this setting has been shown to present more commonly amongst males, younger patients, those having sustained higher velocity injuries, and those with narrower C2 canal size [5,43]. It is noteworthy that the presence of neurological injury is also associated with a high rate of mortality [43].

It has been shown that a large proportion of patients with diagnosed OFs after cervical trauma show evidence of non-acute injury, suggesting the trauma experienced is rather “acute-on-chronic” [46]. Indeed, it has been estimated that up to 40% of geriatric odontoid fractures are not diagnosed [47]. For patients encountered with trauma and in whom previous non-union of an OF is observed, the incidence of neurological injury has been reported at 17.5% [46].

Besides a detailed neurological exam, the initial work-up typically involves radiographs, including lateral imaging and open mouth views which allow for a clear view of the odontoid. However, these may miss non-displaced OFs, and therefore the gold standard radiological exam is a cervical CT scan. If significant odontoid displacement is observed or the patient presents with a neurological injury, it is important and useful to assess the degree of cord compression as well as the integrity of associated cranio-cervical junction ligaments, most importantly the alar and cruciate (notable the transverse component) ligaments [48,49]. It has been reported that the transverse atlanto-ligament is ruptured in ~10% of patients and it has been suggested that if there is >3 mm separation between C1 and the dens, as measured by the atlanto-dental interval (ADI), it is suggestive of transverse ligament injury [49,50,51]. This indicates the presence of potential atlanto-occipital dissociation injuries. Indications for MRI evaluation include assessment of the age of the fracture, assessment of ligamentous injury, and assessment of the spinal cord [7].

## 7. Treatment

The treatment of odontoid fractures can be conservative or surgical, and the choice is dependent on the type of fracture, presence of spinal instability, and patient factors; that is to say, some patients could have radiological and mechanical indication for fixation, but patient factors are not conducive to operative treatment. The aim of all treatment options remains the same and includes spine realignment, stabilization and fusion, and decompression (if needed). Generally speaking, a strong indication for surgical treatment is spinal cord injury and a high risk of non-union, whereas strong contraindications for surgical treatment include patients with limited life expectancy (for example due to advanced dementia), and significant medical risks associated with potential surgical treatment. The various treatment options are discussed in further detail below. It is important to note, that this section focuses mostly on OFs in the elderly population.

## 8. Surgery

When considering isolated odontoid fractures without other associated injuries to the cranio-cervical junction, surgical treatment is generally reserved for patients with OF type II fractures, whereas type I and III fractures are generally treated with non-operative treatment [13]. Particular attention must be paid to type I fractures, because sometimes they are associated with C0–C1 vertical instability or ligamentous injury (e.g., apical and alar ligaments) which require a cranio-cervical junction fusion [7]. In rare cases, type III fractures may also be treated surgically. Typically, this is for cases where type III fractures are higher in the body and shallow, and therefore behave more like type II fractures [52]. According to some authors [53,54,55], an association between conservative treatment of type III OF and non-union with displacement was observed in patients older than 40 years of age, with translation >5 mm, and angulation >10°. Furthermore, some patients who cannot tolerate long-term solid external fixation can be good candidates for surgery. Likewise, some surgeons treat OF type II fractures non-operatively initially, rather than surgically, if no neurological symptoms are associated [56].

Another fundamental variable that should be taken into account is patient age. In fact, there are substantial differences in terms of surgical or non-operative indications for elderly patients and younger ones. In elderly patients, for whom the non-operative treatment may be preferred due to comorbidities, the non-union rates are generally higher with hard collars compared to the younger population, and halo vest fixation is practically abandoned in this population [57,58]. Also, the choice of surgical technique may lead to different results in the elderly. For instance, as highlighted by Texakalidis et al., anterior dens screw fixation showed lower rates of fusion and higher reoperation rates in patients older than 60 years of age. Hence, posterior fixation is preferable for this population [59]. On the other hand, younger patients are more likely male with motor vehicle collision as the mechanism of injury and present generally with more severe trauma, neurological deficit, and other associated lesions. However, the non-union rates are significantly lower at follow-up compared to elderly counterparts. Indeed, external fixation with halo vests can be a viable option for younger patients with low non-union rates [57].

When surgery is undertaken, multiple surgical options exist and are listed in Table 2. It is important to note that for all surgical procedures, wearing a hard collar in the postoperative period might be recommended to increase the fusion rate, even though this measure is controversial. This, however, comes with the risk of hard collars b, which will be discussed further in the non-operative treatment section.

### 8.1. Anterior Approach

The anterior peg screw fixation allows for stabilization of OFs by the insertion of a lag screw across the fracture line. Unlike posterior C1–C2 fixation, it maintains rotation at the level of the C1–C2 joints by restoration of dens anatomy and alignment [59]. The choice for an anterior approach with an odontoid screw is typically reserved for Roy-Camille type IIA and type IIC (Table 1) fractures, where the obliquity of the fracture is perpendicular to the anterior screw insertion. However, it may also be used in Roy-Camille type IIC fractures, where the fractures line is horizontal. The success of the technique requires that the odontoid is reduced and well aligned; this may be aided by intraoperative transoral digital reduction [62]. Sometimes screw insertion is supported with bone cement, particularly in patients with osteoporosis or lytic metastatic disease [61]. In other instances, anterior trans-articular screws may be performed in isolation or may also be added if it is deemed that the odontoid screw fixation is suboptimal, or for a stronger fixation in osteoporotic patients [60]. Minimally invasive approaches to performing anterior screw fixation have been reported as a safe and effective way to achieve satisfactory fusion [63]. However, according to a recent meta-analysis published by Texakadilis et al., anterior dens screw fixation is associated with statistically significantly lower odds of fusion and higher odds of reoperation compared to C1–C2 arthrodesis. This is particularly true for patients older than 60 years [59]. Another meta-analysis performed by Lvov et al. demonstrated that double screw dens fixation in elderly patients results in higher risks of postoperative cut-out. This study showed also that patient age is a major risk factor of fracture non-union and that the fusion rate did not depend on the technique [64].

Anterior screw fixation is contraindicated in patients with transverse ligament injury, as well as for comminuted fractures, and it is important to note that even with a clinical and radiological indication, technical issues may prevent access for anterior screw placement. These include inability to reduce the fracture, or body habitus restrictions, such as a short neck, barrel-shaped chest, or cervical or thoracic deformity [65].

### 8.2. Posterior Approach

Posterior surgery can be undertaken for any of the odontoid fracture types and can be performed in different ways. In 1992, Magerl and Jeanneret described for the first time a technique to realize posterior arthrodesis for odontoid fractures.

The classic Magerl technique consists of placing screws along a trans-articular trajectory, inserting a bone graft in the interlaminar space, and additionally securing it with a wire or hook system. Fully threaded screws are often employed, with the primary reliance on fusion in the interlaminar space. Its advantages include that it requires the placement of only two screws without the need for rods, which reduces operative time. However, it permanently fuses the C1–C2 articulation. It is also important to note that this technique is generally contraindicated in patients with high-riding vertebral arteries because, in these cases, the vertebral artery location is typically more medial and more posterior, and reducing the size of the isthmus increases the risk of injury to the artery during screw insertion [66]. However, techniques for mobilizing the artery to allow for the Magerl trans-articular fixation have been described [66].

**Figure 2:**
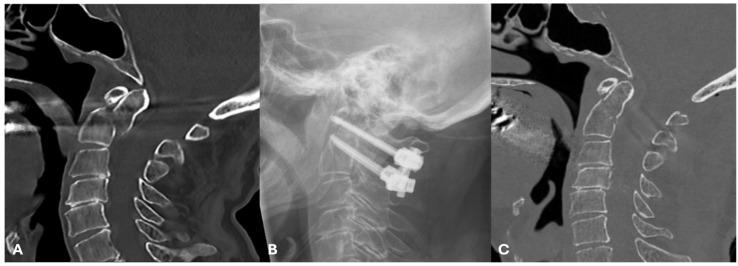
Clinical Case of an Anderson and D’Alonzo type II, Roy-Camille type II odontoid fracture: (**A**) Sagittal CT showing the OF, with some minor posterior displacement of C1 relative to C2, (**B**) postoperative lateral radiograph, showing a posterior C1–C2 fixation using the Harms-Goel technique, and (**C**) postoperative midline sagittal CT of the cervical spine showing good realignment of the odontoid post fixation.

At the present time however, the Goel-Harms technique involving C1 and C2 screws with or without the placement of a bone graft in between the posterior elements of C1–C2 is the most common technique [67,68,69] (Figure 2). This technique represents an alternative to the Magerl technique and involves placement of bilateral C1 lateral mass screws, and C2 pedicle or pars screws, fixed by rods. Some studies have shown that this technique provides the highest rate of union, while others suggest Magerl’s technique is superior. The benefit of the Goel-Harms technique is also that the construct can be removed in younger patients with eventual odontoid union to improve the loss of cervical rotation from fixation [70].

Posterior surgery before the Magerl and Harms Goel techniques involved wiring or clamping and the placement of bone grafts. An alternative to these methods is stand-alone screw instrumentation, which, due to the compressive effect on the lateral masses, leads to their ankylosis. These techniques can be performed percutaneously or minimally invasively through a transmuscular approach [71,72]. Many different variations of wiring techniques have been reported, but this practice has largely been abandoned in developed countries. In countries with limited resources and in situation where screw placement may not be possible, wiring of the posterior element remains an option. However, for these techniques, the posterior arch of C1 needs to be intact [47]. Iyer et al. [47] provides a brief review detailing these techniques.

## 9. Non-Operative Treatment

Non-operative management is a viable treatment option in the majority of isolated odontoid fractures, even type II, which are often treated surgically. Typically, this involves a hard collar or halo vest placement, with the latter becoming rarely used because of its morbidity, especially amongst the elderly. However, even the use of hard collars is not without morbidity [73]. Before the placement of rigid collars, attempts are typically made to align fractures with traction, if necessary. The goal of the non-operative treatment is to achieve a fibrous non-union between the two fragments and therefore a “relative stability” of the odontoid. A systematic review including 714 cases with treatment by halo vest or hard collar showed no significant difference in failure rates, but did show a higher rate of complications associated with halo vests [74], supporting the preferential use of hard collars if external immobilization is sought. Of note, the failure rate with non-operative treatment for type II OFs was higher than for type III (20% vs. 7%), and only a single patient in this review was noted to have neurological deterioration with non-operative treatment.

There is an increased consideration for not placing any immobilization devices in patients treated conservatively, and this has resulted in the formulation of a randomized control trial (DENS) to assess collar vs. no collar treatment [75].

## 10. Outcomes and Complications

The clinical outcome and rate of complications depend on a number of factors, including patient-related factors, the type of fracture, and whether treatment is operative or non-operative. Furthermore, when surgery is performed, the outcomes and complications can vary from anterior and posterior surgery. While many factors can be considered when discussing outcome, the most relevant points related to OFs include rates of union or mechanical stability, wound complications, neck pain, mortality and neurological function. However, it is important to note, as mentioned previously, that short term morbidity and mortality is considerably higher in patients with neurological injury following OFs, regardless of treatment [5,43].

For simplicity, the discussion of outcome and complications is segregated below into two sections: operative vs. nonoperative management and anterior vs. posterior.

### 10.1. Operative vs. Nonoperative Management

Multiple studies have been conducted to assess the relative merits between operative vs. nonoperative treatment; however, the findings of individual studies have been limited by the lack of randomization, which has a significant impact in selection bias in this largely old and fragile population cohort. Systematic reviews [56,76,77,78,79,80,81], have also been somewhat limited in showing a clear efficacy of one treatment over the other. From these reviews, it appears that there may be a slight survival advantage in operatedpatients; however, since there is an absence of RCTs, this may be due to selection bias. A more consistent finding from these reviews is the higher rate of union with surgical treatment. The most recent meta-analysis showed that fracture stability was observed in 82% and 53% of surgically and conservatively treated patients, respectively [80]. However, the importance of non-union is questionable, while a few patients will suffer from chronic pain due to non-union [45], there is a lack of evidence that non-union results in a worse clinical or function outcome in the elderly [56]. Indeed, many of these patients will develop a malunion, or fibrous non-union, without instability objectified on dynamic imaging, and a systematic review on this population supports these fibrous non-union are generally safe and well tolerated by patients [82]. Though union rates are higher with surgery, it has been shown that delay in the initiation of surgery (>3–7 days) is a significant factor influencing non-union [47,83].

It is clear that to definitively determine whether surgery or conservative management is superior, randomized trials are necessary. In 2014, such a study was proposed and registered [84], but no results have yet been reported.

### 10.2. Anterior vs. Posterior Surgery

Although indications for anterior or posterior surgery vary, multiple studies and systematic reviews have been carried out to assess the relative superiority of one surgery over the other [59,85,86]. These reviews indicate that posterior fusion has higher union rates, and lower reoperation rates, than anterior screw placement in patients >60 years [59]. However, there do not appear to be differences in mortality. While the occurrence of adverse events was not shown to be different, it is worthy to note that the types of complication between the surgery types differ. In anterior surgery, as with ACDFs, dysphagia and recurrent laryngeal nerve injury complications may occur, while wound infection is extremely rare, but not uncommon with posterior surgery.

## 11. Conclusions

The number of OFs will continue to rise in populations with aging demographics, and therefore result in an increased health care cost burden. While the management of certain types of fractures are relatively standardized, such as non-operative treatment for type I fractures, the treatment strategy of type II fractures remains variable, with both non-operative and operative treatments being employed, largely due to the heterogenic clinical and radiological variables to consider. Regardless of the treatment, given the advanced age of most patients suffering from OFs, morbidities and mortality are not insignificant, even if optimal treatment strategies are employed. Despite some evidence showing that surgery for type II fractures offers more favorable outcomes, there exists no randomized control studies to support this, and further work in standardizing who benefits most from surgery is necessary.

## Figures and Tables

**Table 1:** Description of Odontoid Fracture Classifications or New Fracture Types.

Publication	Description of Classification, or Type of Fracture
Anderson and D’Alonzo [9]	**Type I** is an oblique fracture through the upper part of the odontoid process itself. **Type II** is a fracture at the junction of the odontoid process with the vertebral body of the second cervical vertebra. **Type III** is a fracture through the body of the atlas.
Roy-Camille et al. [12]	Modification of Anderson and D’Alonzo type II fractures regarding the orientation of the fracture: **Type I**—Anterior inferior to posterior superior **Type II**—Anterior superior to posterior inferior **Type III**—Horizontal line **Type IV**—Comminuted type “English Policeman Hat”
Hadley et al. [11]	**Type II** (Anderson D’Alonzo) fractures that have additional ship fragments (comminuted) fractures, referred to by the authors as Type IIA.
Grauer et al. [13]	The authors modified the Anderson and D’Alonzo classification to better differentiate between type II and type III. They proposed that fractures at the base of the odontoid and involving the body that are shallow and do not involve the superior articular facets should be considered type II, and only fractures of the body involving the superior facets should remain type III.
Vaccaro et al. (AOSpine Classification) [14]	According to the AOSpine classification, all C2 vertebrae and C2–3 joint fractures areclassified as **type III**. Within this group, all isolated odontoid fractures are classified as **Type A.**

**Table 2:** Surgical Techniques for Managing Odontoid Fractures.

Anterior Approach	Technique	Typical Indication
Odontoid Screw fixation [60]	One or two large odontoid screws are placed caudal–cranially in the direction of the odontoid from an anterior cervical approach.	Type IIA and IIC (Roy-Camille) fractures shown in Figure 1.
Odontoid Cement Augmentation/Kyphoplasty [37,61]	In conjunction with anterior screw fixation.	Presence of bone cysts, lytic metastatic fracture of the odontoid, osteoporosis-associated odontoid fracture.
Anterior C1-C2 trans-articular screw fixation [60]	Screws are inserted caudally and slightly lateral to the anterior C1–C2 articular joints. The screw is directed approximately 30° laterally and posteriorly across the C1–C2 joints.	Can be used as an adjunct to odontoid screw fixation when screw fixation is deemed inadequate. It may also be used as a bailout procedure, or due to unfavorable posterior boney anatomy.
Posterior Approach		
C1-C2 Fixation [60]	Either via pars, pedicle, or laminar screws in C2 and lateral mass of C1.	Unstable type II fractures where anterior surgery is not possible or preferred.
C1-C2 trans-articular screw fixation [60]	Screws are introduced at pars of C2 and oriented upwards into lateral mass of C1.	Unstable type II fractures where anterior surgery is not possible or preferred. Usually contraindicated with high riding vertebral artery anomalies.
Fixation C1-C2 without the use of screws [36,47]	Multiple techniques described including: Gallie Wiring Technique Brooks–Jenkins Wiring Technique Halifax Clamping Technique Dickman Wiring Technique	These techniques were previously used for the same indication as for C1–C2 screw fixation, prior to the introduction the latter technique. These remain viable options if there are contraindications to screw fixation or as bailout procedures.

## Data Availability

Not applicable for this article.
